# Chasing elusive expressive writing effects: emotion-acceptance instructions and writer engagement improve outcomes

**DOI:** 10.3389/fpsyg.2023.1192595

**Published:** 2023-06-13

**Authors:** Stephanie S. Rude, Crystal Lantrip, Vanessa A. Aguirre, William A. Schraegle

**Affiliations:** ^1^Department of Educational Psychology, The University of Texas at Austin, Austin, TX, United States; ^2^Center of Excellence for Research on Returning War Veterans at Central Texas Veterans Health Care System, Waco, TX, United States; ^3^Department of Psychology and Neurosciences, Baylor University, Waco, TX, United States; ^4^Department of Psychology, The University of Texas at Austin, Austin, TX, United States; ^5^Department of Neurology, Dell Medical School, The University of Texas at Austin, Austin, TX, United States

**Keywords:** expressive writing, emotion acceptance, moderators, engagement, intervention, depression

## Abstract

**Introduction:**

Pennebaker’s expressive writing (EW) paradigm in which participants are encouraged to explore their “deepest thoughts and feelings” about a difficult experience in several short writing sessions has yielded impressive mental health outcomes and holds great promise as a cost-effective intervention. Yet results have been difficult to replicate and it is unclear what conditions are necessary for observing the effect. Our aim was to discover reasons for the variability in EW outcomes. We explored the impact of augmenting writing instructions to encourage acceptance of emotional experience, which we thought would encourage engagement with writing; and we examined essay length, an index of writer engagement, as a possible moderator of writing outcomes.

**Methods:**

We compared traditional expressive writing (tEW), conducted according to Pennebaker’s paradigm in which participants write about a self-chosen emotional experience for 15 min at a time on each of three closely spaced days, with an acceptance-enhanced version (AEEW), identical except that it supplemented traditional instructions with encouragement of an accepting approach to emotional experience, and with a control condition which asked participants to write about their use of time on particular days. Self-reported depression was the outcome measure.

**Results:**

Essay length (a proxy for writer engagement) moderated effects of writing at posttest 2 weeks later: Condition differences were found only for participants who wrote longer essays: For these participants the AEEW condition outperformed both control and tEW; and tEW did not differ significantly from control.

**Conclusion:**

Findings suggest that degree of engagement in the writing process may partially explain the puzzle of variable outcomes in the EW literature. Results also provide practical guidance: those who are motivated to engage deeply in the writing process are most likely to benefit; and encouraging writers to accept and to openly explore emotional experience is expected to enhance benefits.

## Introduction

In 1986, Pennebaker and Beal published their seminal study demonstrating that brief sessions of writing about emotions produced physical and mental health benefits (Pennebaker and Beall, 1986). In the classic paradigm developed by Pennebaker and colleagues, participants are instructed to write for 15–20 min across three consecutive days, exploring their “deepest thoughts and feelings” about an important personal and emotional topic without worrying about grammar or punctuation. The efficacy, brevity, and cost-effectiveness of this intervention has fueled excitement and contributed to an explosion of research. Enthusiasm has stemmed not only from the effectiveness of this brief intervention, but also from the theoretical support, it provided for the value of exploring emotions.

A large literature documents the successes of EW. But the effect has proven elusive, with three meta-analyses supporting the benefits of writing about emotions and three others yielding null effects: Smyth (1998) examined 13 studies using healthy samples and found a medium sized effect for psychological health (e.g., depression) and for physical health, measured both directly (e.g., blood pressure) and by self-report (e.g., number of doctor visits). Frisina et al. (2004) extended these findings in a meta-analysis of nine studies using clinical samples of individuals selected for either physical or psychiatric disorders. They found a small effect for a group of physical health measures (physiologic and self-report), as well as for several of the psychological outcomes examined (mood, depression, anxiety, and sleep quality). In a third and particularly comprehensive meta-analysis, Frattaroli (2006) reviewed 146 studies using both healthy and clinical samples and a variety of outcome categories including self-reported health (e.g., number of doctors’ visits), objective health indices (e.g., HIV viral load), and self-reported psychological symptoms (e.g., depression). Frattaroli found a small but significant effect of EW for these outcomes.

In contrast, several recent meta-analyses have failed to find significant effects for EW. Meads and Nouwen (2005) analyzed physiologic and psychological outcomes across 61 studies and found no effects. Mogk et al. (2006) analyzed psychological and physical health variables across 30 studies and found no effects. Most recently, the meta-analysis of Reinhold et al. (2018) failed to find a benefit of EW in 39 studies using self-reported depression symptoms as outcomes (It should be noted that this meta-analysis excluded studies using samples with PTSD and focused on results at follow-up).

Collectively, the three meta-analyses yielding null results have challenged prevailing wisdom about the effectiveness of EW. Most importantly, it has become clear that we do not know under which circumstances benefits can be expected to occur, a problem we must resolve if EW is to be of use for treatment purposes.

Many researchers have responded to this puzzle by looking for moderators—differences in attributes of respondents or in the way EW is conducted—that may explain the inconsistencies. Unfortunately, results of these efforts have been inconsistent and sometimes directly contradictory (see Rude and Haner, 2018). Even across meta-analyses, conclusions regarding moderators have not been consistent. For example, Reinhold et al. (2018) found that samples with larger proportions of female participants reported lower depression following EW; but Smyth (1998) found evidence for the opposite pattern in psychological well-being measures, and Frattaroli found no effect of sex on any outcomes. Reinhold et al. (2018) reported better depression outcomes in older samples but Frattaroli (2006) and Smyth (1998) both found no effect for age on any outcome category. Relevant to the current study, the meta-analysis of Frattaroli (2006) found that effects on measures of psychological well-being were marginally smaller in studies using college sample vs. non-college samples, but Smyth (1998) found the opposite pattern.

Frattaroli (2006) examined a number of possible moderators that other meta-analyses did not. In these analyses, Frattaroli (2006) found stronger reported health benefits in study samples with high stress levels, and stronger psychological well-being effects and marginally stronger reported health effects for participants low in optimism. It should be noted that these analyses were based on very few studies.

Frattaroli (2006) also found evidence for several procedural moderators. Better outcomes were associated with studies that used three or more writing sessions, sessions lasting at least 15 min, writing instructions providing more specific directions, and writing at home or in a private space. Reinhold et al. (2018) reported similar findings for session length and the number of writing sessions, and also found that longer spacing between writing sessions was beneficial. Moderation analyses of other procedural variations between studies were not reported in meta-analyses other than Frattaroli’s. However, most EW studies have used methodologies consistent with these procedural moderators and they do not sufficiently explain variability in the outcomes found in the literature.

One possible moderator of EW results that has received surprisingly little attention but that may have powerful effects is the degree to which participants engage deeply in the process of writing. As Rude and Haner (2018) have pointed out, there are numerous reasons why participants may not be motivated to engage deeply in EW. Instructions ask respondents to grapple with challenging emotional states and call for a high degree of effort and emotional vulnerability.

This is important because degree of engagement with writing seems likely to be central to the effectiveness of EW in much the same way that engagement in the process of psychotherapy is key to its success. Within the psychotherapy literature, there is evidence that greater experiencing of anxiety (signifying deeper engagement) facilitates successful treatment of anxiety disorders (Foa, 1997; Jaycox et al., 1998; Keefe et al., 2019) and that emotional intensity strongly predicts outcome across varying treatments of depression (Beutler et al., 2000).

Although depth of engagement in EW has not, to our knowledge, been studied systematically, there is some evidence that it is important for positive effects of emotional disclosure. For example, Lepore and Greenberg (2002) found that higher proportions of negative emotion words (e.g., “sad”) used in writing prospectively predicted improvement in physical health from baseline to follow-up. Similarly, Lutgendorf et al. (1994) found that depth of emotional experiencing during verbal disclosure sessions (as rated by interviewers) and decreased avoidance across disclosure sessions (self-reported) predicted positive immunity outcomes; and Sloan et al. (2022) found that expressing negative emotion predicted improvement in each of two exposure-based treatments for PTSD, one of which was a written treatment, similar to EW. Finally, O’Cleirigh et al. (2003) coded essays written by HIV positive individuals and found that emotional expression and depth processing were related to long-term survival.

### Present aims

The present study pursued variations in engagement with the writing process, as indexed by essay length, as a possible reason for the elusiveness of EW effects. We also tested the effectiveness of an acceptance-enhanced condition (AEEW) in which traditional EW instructions were augmented by statements normalizing distressing emotions and encouraging open exploration of them. We compared this condition to one using traditional EW (tEW) instructions, as well as to a control (writing about time management). We hypothesized that the AEEW instructions would produce greater benefits for depression symptoms as compared to traditional or control instructions. Baum and Rude (2013) found that a condition very similar to the AEEW condition used in the present study was superior to a control condition in mitigating depression symptoms whereas no effect was found for a standard EW condition. As for the mechanisms of effect, we reasoned that these instructions would motivate participants to engage more deeply with their challenging emotions by reducing threat and self-judgment. But it is also plausible that these instructions might enhance the effectiveness of EW by altering the way participants reappraise and make meaning of their problems (*cf.*, Lepore et al., 2002).

Essay length was used as a proxy for degree of engagement in the writing process, and we hypothesized that benefits of EW would be greater among participants who wrote longer essays. We used self-reported depression as an outcome measure because even subclinical levels of depression represent an important mental health problem (e.g., Judd, 1995), and depression has been commonly used in EW studies.

## Methods

### Participants

Data were collected for the purposes of another study (Rude, 2022) over the course of three semesters from the Educational Psychology research pool at the University of Texas at Austin. The only requirement of the study was that participants be 18 years or older. More details are provided under Procedure below.

Sessions 1–4 were completed by 970, 930, 902, and 862 individuals, respectively. There were 18 cases lost because we were unable to connect data from Session 4 with all prior sessions. This occurred because we relied on a code that participants were instructed to create during each session to connect data (see the section Procedure) and there were a number of ways to err in reporting this code. In addition, 11 cases were eliminated from the dataset because these participants had completed one or more of the sessions twice.

In the final sample of 833 participants, 67% were female, and the mean age in years was (*M*_age_ = 20.76, *SD* = 2.26). Participants reported various ethnic backgrounds, with 6.5% Black/African American; 19.6% Hispanic/Latino; 0.4% Native American; 19.3% Asian American; 3.2% South Asian American or Pacific Islander; 43.0% White/Caucasian; 2.3% Middle Eastern/Arab American; 4.0% Biracial or Multiracial; and 1.8% Other. Roughly, 80% of the sample endorsed English as their primary language; however, of those for whom English was a later-learned language, 89% rated English to be easy for them. No significant differences emerged between assigned writing conditions and age, gender, ethnicity, English facility, or initial depression symptoms.

### Measures

#### Center for Epidemiological Studies Depression Scale-10-item

This is the shortened version of the 20-item Center for Epidemiologic Studies Depression Scale (CES-D), which is one of the most common self-report measures for identifying depressive symptoms in the general population (Radloff, 1977). For each item participants respond with how often they have felt this way during the past week, from “Rarely or none of the time (Less than 1 day)” to “Most or All of the Time (5–7 days).” Items include “I was bothered by things that do not usually bother me” and “I felt fearful.” Internal consistency is high for the CES-D-10, with researchers reporting a Cronbach’s alpha of 0.86 (Miller et al., 2008).

#### Visual Analog Mood Scale: Energetic Subscale

This subscale is a brief measure of positive mood, a subscale of the Visual Analog Mood Scale (VAMS; Stern et al., 1997). Participants were asked to indicate their current mood by moving a bar along a horizontal line, with “I feel the worst I have ever felt” on the very left and “I feel the best I have ever felt” on the right.

#### Self-rating scales and open-ended comments

Scales and comment text boxes were included to query participants’ self-rated involvement and experience of the study. These were preceded by the statement: “It is very helpful for us to know how participants have approached the study, and so your frank answers are appreciated.”

Likert style questions were used to assess self-reported honesty and accuracy in completing questionnaires: “How honest/accurate were your responses to the questionnaire items?” and, “Do you feel you were able to follow the writing instructions fairly well?” Finally, participants were given the prompt: “Do you have any comments regarding the way you followed instructions or the way you responded to the study? We welcome your thoughts.” The Likert style questions were answered on a five-point Likert-style scale ranging from “not at all” to “very much.”

### Procedure

Participants completed a total of four online sessions on a secure server from a location of their choosing. They accessed the first study session by visiting the departmental research pool website and choosing from a number of studies that would count toward fulfillment of their course research requirements. Participation was anonymous: Participants were guided to use a code (first three letters of mother’s first name followed by the month and date of their own birthday) that was used to link the data from the four sessions. Partial credit was given for completion of each session.

In the first session, participants completed a number of questionnaires including the questionnaires described above as well as several not relevant to the current study. Participants were then randomly assigned to one of three writing conditions and asked to write for 15 min. The screen could not be advanced until the 15 min had passed, and once it had, a timer notified participants that they could stop. Immediately after this and the other two writing sessions, participants indicated their current mood using the Visual Analog Mood Scale described above, and completed a self-rating scale (described above) to indicate how well they followed writing instructions. At the end of the first and the final sessions (in both of which the CES-D was administered), participants used self-rating scales (described above) to indicate how honestly and accurately they had responded to questionnaires.

The second and third writing sessions (both in the originally assigned condition) were completed over the next several days: One day (24 h) after completing each of the first two sessions, participants received an emailed link to the subsequent session, and were asked to complete it within the next 48 h. Two weeks after they had completed Session 3 (the final writing session), participants received the link for Session 4, which consisted of the CES-D and other post-intervention measures not relevant to current purposes, and were asked to complete these within 48 h.

#### Writing conditions

Participants were randomly assigned to either tEW, AEEW, or a time management control. Instructions for each condition at all three sessions ended with the direction to write continuously for the full 15 min as well as a reminder that all writing would be completely confidential. Next, participants were directed to a blank screen to write.

##### Traditional expressive writing instructions

Instructions were those developed by Pennebaker (1989, 1997) and used in numerous studies. Participants were first encouraged to think about a writing topic for the session:

“Today and for the next two days, you will be asked to write about your very deepest thoughts and feelings about an extremely difficult or emotional event that has affected you. You will receive further instructions but first please take a moment to think of a situation to write about. Think about when the event occurred and what you were doing.”

Next, participants were given the classic instructions originally developed by Pennebaker (1989, 1997).

“In your writing try to really let go and explore your very deepest emotions and thoughts. Feel free to write about any aspect of the difficult situation and the way you feel about it that comes to mind. Don't worry about spelling, sentence structure, or grammar. The only rule is that once you begin writing, continue to do so until your time is up. Remember, all of your writing will be completely confidential. Begin writing. Let go and explore your very deepest emotions and thoughts. Please keep writing for the entire 15 minutes!”

##### Acceptance-enhanced expressive writing instructions

The acceptance-enhanced expressive writing (AEEW) condition was based in prior work by Rude and colleagues that showed greater benefit when requests to write emotionally were accompanied by instructions emphasizing the universality of distress and openness toward emotional experience (Rude et al., 2011; Baum and Rude, 2013). The enhanced EW instructions included the instructions shown above but these instructions were prefaced by the following message intended to increase participant acceptance of and willingness to explore challenging emotions.

“Before writing, please take a moment to notice your feelings related to the emotional event you’ve chosen. When people go through difficult events they may experience emotions such as shame, rage, jealousy, resentment, anxiety, sadness, and embarrassment. Sometimes these emotions include physical reactions such as, racing heart, sweaty palms, upset stomach, and tears. Often people try to avoid these experiences and feel ashamed of painful emotions. Although your impulse may be to push your distressing emotions away, try to bring a curiosity to your experience and be accepting of any emotions or thoughts that arise. Emotions come and go for everyone--they are not permanent. And even unpleasant emotions often lead to learning and growth. Think about how you would react to a close friend experiencing these things. Try to express the same sort of kindness and understanding towards yourself as you would towards someone you are close to. Remember, we all go through difficult feelings and distressing events—you aren't alone in your experience.”

##### Time management control instructions

The control condition was modeled after a time-management control used by Pennebaker and colleagues in previous studies (e.g., Pennebaker and Beall, 1986; Pennebaker et al., 1988, 1990). In order to make these instructions more face valid, instructions were varied slightly between each session so that they referenced a past or future day and referred either to how a participant had used their time or *planned* to use their time on that day.

“In your writing for today, please describe in detail how you used [or plan to use] your time ON THIS DAY you have chosen. In your writing, please be as objective as possible. For the purpose of the exercise, try not to be distracted by your emotions or opinions. Rather stay as close to the facts of how you used [or plan to use] your time and be as specific as possible. Describe what you did [or plan to do] on your chosen day, from the time you got up until the time you went to bed. Remember, all of your writing will be completely confidential. Begin writing. Describe in detail how you used your time on this particular day.”

#### Data analytic procedures

**Figure 1:**
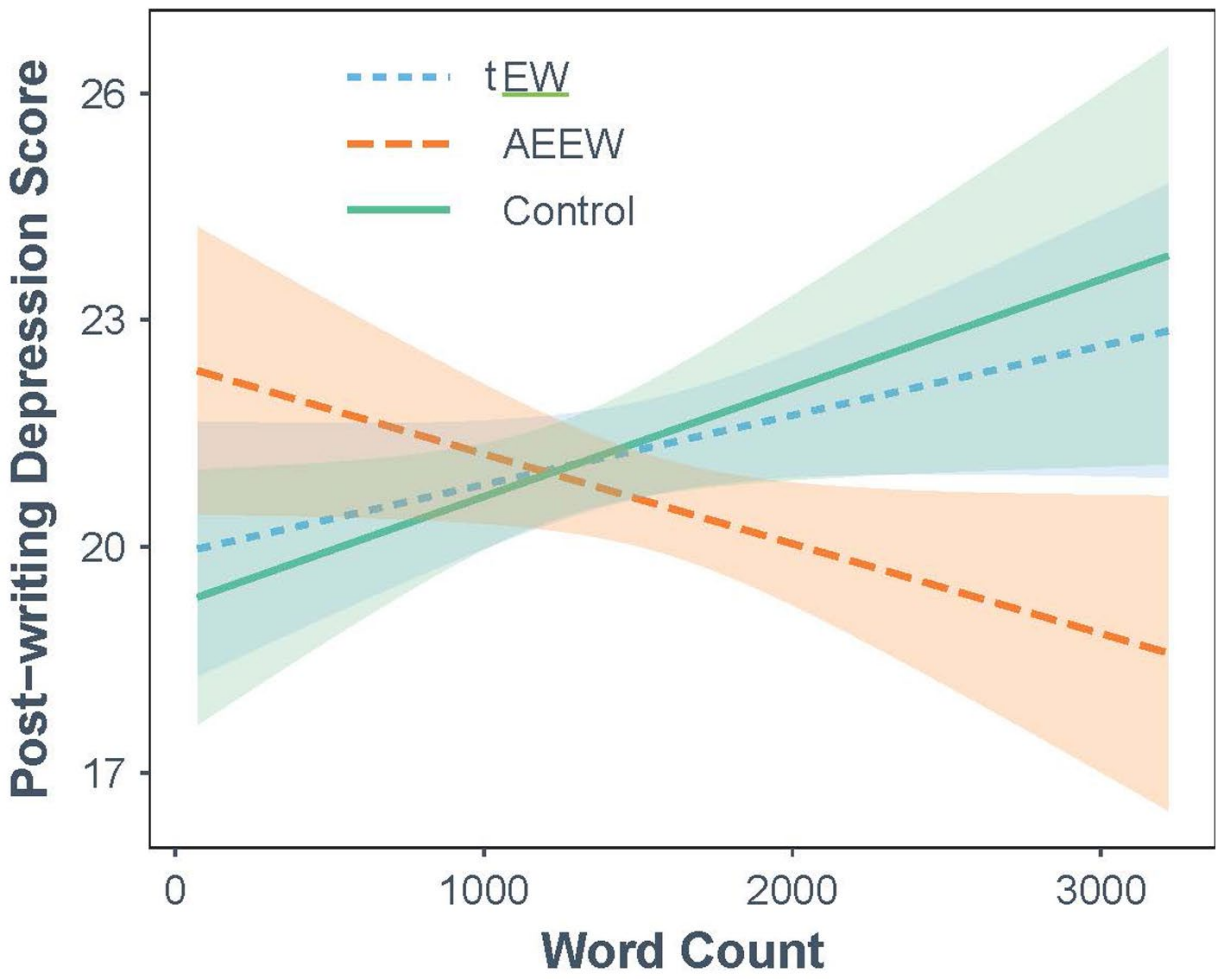
Effects of essay length and writing condition on post-writing depression scores.

SPSS version 27 was used to calculate summary information about mean pre-and post-depression scores and counts of positive and negative words as well as overall word counts. R version 1.3.1093was used to conduct Johnson-Neyman confidence bands and generate Figure 1.

##### Manipulation checks for writing condition

The Linguistic Inquiry and Word Count program (LIWC; Pennebaker et al., 2001) was developed for the purpose of counting word usage percentages from written texts in a large number of categories (e.g., pronouns, past, present, and future tense words, and emotion words) and was used here to derive counts of the overall number of words written by each participant, as well as the percentage of positive and negative words used by each participant.

Comparisons of the three study conditions were made using one-way ANOVA with *post hoc* contrasts using Tukey’s Least Significant Difference tests (Tukey, 1949) wherever the omnibus F ratio was significantly different from zero. The conditions were compared on proportions of emotion words (both positive and negative), essay length, self-reports of adherence to instructions, and mood ratings for the purpose of checking the writing instruction manipulation. Mood ratings made immediately following each writing episode were compared between conditions because EW has consistently been associated with more negative mood in the immediate aftermath of writing and has sometimes been attributed to the process of grappling deeply with unpleasant memories (Pennebaker, 1993; Lepore et al., 2002).

##### Checks on the validity of treating essay length as an index of engagement

In order to substantiate our assumption that essay length functions as an index of engagement in the writing process, we examined its correlation with three other indices of engagement: First, we used participants’ self-ratings of how closely they had followed writing instructions. We examined the correlation of post-writing ratings (summed) with word count (also summed across the three writing episodes). Second, we examined the correlation between essay length and mood ratings made immediately after writing (scores for both variables summed across writing sessions) on the logic that negative mood at this point appears to reflect engagement (see Lepore et al., 2002; Lepore and Greenberg, 2002).

Third, we examined the correlation between essay length and a qualitative index of engagement with writing. As indicated under Methods, participants had been invited to comment at the end of each writing session about “… the way you followed instructions or the way you responded to the study” Responses to this very open-ended question were optional and we reasoned that volunteering commentary about the process or experience of writing would indicate involvement or engagement. For convenience, we compared only the shortest and longest 20% of essays. We counted the number of participants in the top and in the bottom 20% of essay length who mentioned the process or experience of writing and compared the proportion who made such comments using a chi square test. Comments that were counted ranged from statements about emotions that arose during or in the aftermath of writing (e.g., “This was unexpectedly cathartic and tearful…”) to statements about how the writing task was approached (e.g., “I just let my thoughts flow freely…”).

## Results

### Descriptive data

The mean pre and posttest CES-D scores were 20.73 (*SD* = 5.89) and 20.94 (*SD* = 6.35), respectively. CES-D scores at pretest were not statistically different between conditions (*p* > 0.6).

Word counts (summed across the three writing episodes and then averaged across participants) for the tEW, AEEW, and Control conditions, respectively, were 1520.06 (SD = 585.75), 1575.61 (SD = 531.39), and 1230.15 (SD = 464.63). Word counts were significantly lower for the control compared to each of the active conditions (*p*s < .001) but the two active conditions were not significantly different from each other (*p* > .2).

Mean lag times between the first and second sessions and between the second and third sessions, respectively, were 38.05 h (SD = 16.18) and 40.21 h (SD = 16.41). The mean lag time between the third and fourth sessions (designed to have a 2-week lag) was 14.78 days (SD = 2.96). These lag times did not differ significantly as a function of condition, *p* > .05.

### Manipulation checks

#### Mood and use of emotion words by condition

The sum of participants’ mood ratings made immediately after each writing session was lower (more negative) for each of the two active conditions as compared to control (*p*s < .001). Mood ratings for the two active writing conditions did not differ from each other (*p* > .4).

Participants in both active conditions used more emotion words–both positive and negative–than did those in the control (all *p*s < .001). Participants in the AEEW condition used a higher percentage of negative emotion words (*p* < .001) and a lower percentage of positive words (*p* < .01) than did those in the tEW condition.

Participants in the AEEW condition reported following writing instructions (summed across sessions) more closely than did those in the control condition, *p* < .02, but not better than those in the tEW condition, *p* > .5. Ratings of how closely writing instructions were followed in the tEW condition were higher but not significantly different from those in the control condition, *p* > .07.

#### Correlates of essay length

The correlation between essay length and self-ratings of how closely writing instructions were followed (averaged across the three writing sessions for both variables) was positive and statistically significant. *r* = 0.20 (*p* < .001). The correlation between essay length and post-writing mood ratings (averaged across the three writing sessions for both variables) was −0.16, *p* < .001. More negative mood was associated with longer essays. This correlation remained nearly the same (*r* = −0.15) when initial depression scores were partialled out, despite the substantial correlation of initial depression and average mood after writing (*r* = 0.35).

Essay length was also associated with a qualitative index of engagement with writing, examined for participants in the top and bottom 20% of essay length (at or above 1,881 words and at or below 962 words, respectively). Among the 168 participants who wrote the longest essays, 34% of respondents volunteered at least one comment about the process of writing, whereas only 14% of the 166 participants who wrote the shortest essays made such comments. This difference was statistically significant, chi square (1 *df*) = 8.33, *p* < .005.

### Primary analyses

To begin, we examined the main effect of condition on post-test depression scores with initial depression scores as a covariate. The analysis of covariance (ANCOVA) demonstrated no main effect of condition on post-test depression (*p* = 0.21, partial η^2^ = 0.004). Estimated marginal means for post-test depression ratings (CES-D) across conditions were as follows: tEW condition = 21.36, SE =0.30; AEEW condition = 20.68, SE = 0.28; and control condition = 20.80, SE = 0.29.

Next, to address our hypothesis that condition outcomes are moderated by degree of engagement, we assessed the influence of word count in moderating effects of writing condition on post intervention depression scores, while covarying initial depression scores. Results of the two-way ANCOVA demonstrated a significant interaction between condition and word count on post intervention depression [*F* (2, 826) = 4.12, *p* = 0.017; partial η^2^ = 0.010]. The Johnson-Neyman technique (Preacher et al., 2006) was used to estimate regions of significance for which post intervention depression scores differ by condition at different ranges of word count. Three simple contrasts were performed: tEW vs. Control condition; AEEW vs. Control condition; and tEW vs. AEEW.

These contrasts showed that the AEEW condition performed significantly better than the control condition for essay length of 1,722 words or greater (top 29%; *p* < .01). In addition, when essay length was greater than 1,725 words (top 29%), the AEEW condition performed significantly better than the tEW condition (*p* < .05). No differences between the tEW and control conditions were identified across essay length. Figure 1 shows these relationships graphically, depicting the prediction of post intervention depression scores for each condition as a function of essay length (word count). Error bands are shown as lightly shaded areas of color corresponding to each condition. The point at which error bands separate depicts the point along the word count continuum at which condition differences become statistically significant.

## Discussion

The goal of this study was to determine whether depth of engagement in writing (essay length), moderated the EW effect and whether the addition of encouragement to accept and explore challenging emotions, as occurred in the AEEW condition, would enhance tEW benefits. Identifying moderators of EW is important not only because it can account for puzzling mixed results in the literature and can support the veridicality of the effect, but because it can clarify the conditions necessary for success.

As happens in many EW studies, our analyses of main effects failed to find differences between any of the conditions. However, we did find a significant interaction between condition and essay length on post-intervention depression: Among those who wrote the longest essays (approximate top third), the AEEW condition outperformed the control and the tEW condition. As can be seen in Figure 1, tEW did not differ significantly from Control for any range of essay length, and none of the conditions differed from each other among those who wrote shorter essays. The absence of significant condition differences was seen among those who wrote essays below about 1,725 words, roughly the bottom 2/3 of the sample. It seems that both writing longer essays, which we think reflected a greater tendency to engage with writing, and receiving acceptance-enhancing instructions, were key to finding benefits of EW for depression symptoms.

Our interpretation that depth of engagement moderated EW effects is consistent with findings showing increased benefit of engagement in oral (Lutgendorf et al., 1994) and written (Lepore and Greenberg, 2002; Sloan et al., 2022) disclosure. And we think writer engagement has the potential to explain considerable variance in the success of EW interventions. While motivational differences may affect any type of psychological study, we surmise that the effects on EW studies are especially pronounced because EW is effortful and emotionally demanding. In convenience samples (e.g., those comprised of college students), many participants may be unmotivated to engage in effortful, emotionally intense processing. Consistent with this notion, the most comprehensive meta-analysis published to date (Frattaroli, 2006) found that college students were marginally less likely to show benefits of EW on measures indicating psychological well-being (although Smyth, 1998 found opposite results in his small meta-analysis).

But it is also likely that many individuals with high levels of psychological distress are reluctant to engage deeply with unpleasant thoughts and memories as well. For these participants, such experiences may be habitually avoided, and grappling with painful content may feel overwhelming. The two meta-analyses that examined outcome differences for clinical versus non clinical samples did not find moderation; however, the meta-analysis of Frisina et al. (2004), which examined samples with physical health versus psychiatric problems, found stronger EW effects for the former, which could reflect avoidance patterns in psychiatric populations.

In making sense of the current results, it is important to examine a key assumption of the moderator analysis presented here, which is the idea that essay length reflects the depth of writers’ engagement with their thoughts and emotions. On the one hand, several pieces of evidence support this assumption: Essay length was positively and significantly correlated with likert ratings of how closely writing instructions were followed and with negativity of mood immediately following writing. The finding for mood is compelling because negative mood has consistently been shown to be more negative following expressive as compared to control writing (Pennebaker, 1989, 1993) and has been interpreted as reflecting immersion in unpleasant thoughts and emotions (Pennebaker, 1993; Lepore et al., 2002). In addition, essay length was associated with the number of comments participants volunteered about the process or experience of writing. On the other hand, it must be acknowledged that these associations were modest and that essay length is probably a fairly rough index of depth of engagement. Note that we do not know whether essay length, in and of itself, was key to obtaining benefits or whether it was simply an index of the attitude, with which writing was approached.

Another aspect of the current findings that merits discussion is the fact that, among those who wrote the longest essays, the AEEW condition performed significantly better than both the control and the tEW condition. This finding is similar to one reported by Baum and Rude (2013). In that earlier study, writing instructions similar to those used in the AEEW condition but not traditional EW instructions mitigated depression symptoms as compared to a control condition, but the difference between the acceptance-augmented instructions and the traditional EW instructions was not statistically significant. As in the present study, Baum and Rude (2013) also found that participants in their acceptance-enhanced condition used a higher proportion of negative emotion words than the other conditions.

It seems likely that the augmented instructions of the AEEW condition invited participants to engage deeply by normalizing the experience of difficult emotions and indicating the value of exploring them. The greater use of negative emotion words in the AEEW condition supports this interpretation. Using a higher proportion of negative emotion words would seem to suggest greater exploration of challenging situations and experiences and lower levels of avoidance, which offers fairly compelling support for the idea that AEEW instructions facilitated greater engagement. While the fact that essays were not significantly longer in the AEEW as compared to the tEW condition seems not to be consistent with this interpretation, essay length may well not have a completely linear relationship to writer engagement. Essay length may have a threshold effect—that is, a certain essay length may indicate a sufficient degree of writer engagement to produce benefit, and even longer essays may not reflect greater engagement. Or, similarly, engagement may only be beneficial up to a certain point.

It is also true that AEEW instructions may have other effects that account for part or all of their effectiveness. For example, they may alter the way participants construe, appraise, and make meaning out of the experiences they write about. Such a possibility is consistent with the results of studies in which manipulations of the way individuals think about challenging experiences affect psychological well-being. For example, Watkins and colleagues have shown that encouraging individuals to think about unpleasant events at more concrete vs. abstract level reduces self-reported depression (Watkins and Moulds, 2005; Watkins et al., 2008), and Kross and colleagues have shown that encouraging a more self-distanced as compared to immersed perspective has similar benefits (Ayduk and Kross, 2008; Kross and Ayduk, 2008).

While we predicted that the AEEW condition would outperform tEW, the complete absence of effects for the tEW condition was not expected. Given that the best estimates of the size of the EW effect suggest that it is small, this null finding may also reflect low levels of engagement. The current sample may have been low in engagement due to the relatively impersonal context of a fully online study, and may have failed to meet the threshold for effects because of this. In other words, we might have seen tEW effects had our study procedures somehow invited engagement more strongly (e.g., perhaps through more personal interaction with researchers or more engaging descriptions of the research) *or* if our sample had been more motivated (e.g., perhaps by having greater need for intervention or being less preoccupied by academic assignments). In such a case, we would still predict a stronger effect for the AEEW condition and moderation by engagement (essay length).

It should be noted that the use of a sample with variable and fairly low overall levels of engagement does not render the current results less informative, as it is important to establish boundary conditions for observance of the EW effect. Degree of engagement in EW likely varies widely from study to study due to a variety of contextual and individual factors. Our finding that engagement, operationalized here as essay length, moderates benefits from EW interventions, offers a compelling possible explanation for much of the observed variability of results across studies. Our hope is that clinicians may be able to use assessments of engagement to determine who is likely to benefit from EW and that in many cases we may be able to increase engagement as a way to maximize benefits from writing.

While the size of the differences between benefits obtained from the AEEW versus control or tEW conditions among longer essay writers are modest, the fact that significant differences among participants writing longer essays were observed a full 2 weeks after intervention enhances their clinical significance. Many events in the life of a college student can intervene across 2 weeks to influence depression symptoms; the fact that effects of this randomly assigned condition were discernable over this period is notable. And, as has been previously noted (c.f., Frattaroli, 2006), small effect sizes for EW are quite impressive in light of the brevity, portability, and inexpensive nature of this intervention.

### Strengths and limitations

Strengths of this study include the use of a large sample that was fairly ethnically diverse, with over half self-identifying in categories other than white/Caucasian, and about one fifth speaking English as a second language. Another strength was the longitudinal design, with the depression outcome measure being administered about 2 weeks following the end of writing. Further, the fact that responses were anonymous and did not involve face to face contact with experimenters may have reduced demand effects.

An important limitation is that depth of engagement was assumed to be indexed by essay length. While this assumption was supported by associations between essay length and mood, self-reported adherence to instructions, and by volunteered comments about the experience of writing, it is an admittedly crude measure of engagement. Further, engagement/essay length was observed *post hoc* rather than experimentally manipulated, leaving open the possibility that a variable correlated with essay length such as verbal fluency, rather than engagement, moderated benefits from writing. Relatedly, the superior performance of the AEEW condition, while important in and of itself, is not definitively due to its impact on writer engagement. In fact, it is conceivable that the simple fact of instructions in this condition being roughly twice as long as instructions for the other two conditions could be responsible for the effects. And, as discussed above, it is plausible that positive impacts of this condition derive from other effects of these instructions besides engagement. In addition, our use of a single outcome measure, self-reported depression, may not adequately capture changes experienced by participants from writing.

### Implications for future research

One of the proximate next steps for research into EW is to re-analyze existing EW datasets to see if a moderating effect of essay length, such as that found here, can produce similar results. In studies that found EW effects, we would expect effects to be strongest among those who wrote longer essays; and in studies without significant condition differences, the effect could emerge among those who wrote longer essays. While many existing datasets will not be large enough to support such an analysis, many will be.

A particularly important agenda for future research will be to build on the current results by *experimentally* manipulating participants’ motivation to engage deeply with writing. This will be essential toward supporting the interpretation that depth of engagement is causally related to EW benefits, as opposed to alternative explanations such as the possibility that essay length is a byproduct of some pre-existing variable that allows individuals to benefit from EW. It will also be important to directly measure the degree to which participants found the rationale for the conditions compelling and the degree to which they exerted effort and felt immersed in the writing. In this vein, there is a need to develop good measures of writers’ depth of engagement in writing.

The question of how to enhance participant engagement in the writing process is a key and an important agenda for future research. We do not yet have a sufficient research base to guide the process; however, there are places to begin in increasing engagement. It makes logical sense that selecting individuals who have an expressed need for intervention and offering them a compelling rationale and a supportive context for intervention would be important.

## Data availability statement

The raw data supporting the conclusions of this article will be made available by the authors, without undue reservation.

## Ethics statement

The Institutional Review Board of The University of Texas at Austin approved the protocol for the current study (#2018-01-0099). The participants provided their informed consent to participate in this study.

## Author contributions

SR conceptualized and designed the study in consultation with CL and took the lead in writing the manuscript but all authors contributed to the writing and editing of the manuscript. VA assisted with data collection and file management. WS analyzed the data in consultation with SR and CL and wrote up the results. CL and VA reviewed the literature. All authors contributed to the article and approved the submitted version.

## Funding

CL acknowledges the support of the VA from Clinical Science Research and Developmental Career Development Award 1 IK2 CX002101-01A2.

## Conflict of interest

The authors declare that the research was conducted in the absence of any commercial or financial relationships that could be construed as a potential conflict of interest.

## Publisher’s note

All claims expressed in this article are solely those of the authors and do not necessarily represent those of their affiliated organizations, or those of the publisher, the editors and the reviewers. Any product that may be evaluated in this article, or claim that may be made by its manufacturer, is not guaranteed or endorsed by the publisher.
